# A Unique Family of Neuronal Signaling Proteins Implicated in Oncogenesis and Tumor Suppression

**DOI:** 10.3389/fonc.2019.00289

**Published:** 2019-04-17

**Authors:** Markus Hartl, Rainer Schneider

**Affiliations:** Center of Molecular Biosciences (CMBI), Institute of Biochemistry, University of Innsbruck, Innsbruck, Austria

**Keywords:** GAP43, MARCKS, BASP1, Wilms tumor suppressor protein 1 (WT1), MYC, calmodulin (CaM)

## Abstract

The neuronal proteins GAP43 (neuromodulin), MARCKS, and BASP1 are highly expressed in the growth cones of nerve cells where they are involved in signal transmission and cytoskeleton organization. Although their primary structures are unrelated, these signaling proteins share several structural properties like fatty acid modification, and the presence of cationic effector domains. GAP43, MARCKS, and BASP1 bind to cell membrane phospholipids, a process reversibly regulated by protein kinase C-phosphorylation or by binding to the calcium sensor calmodulin (CaM). GAP43, MARCKS, and BASP1 are also expressed in non-neuronal cells, where they may have important functions to manage cytoskeleton architecture, and in case of MARCKS and BASP1 to act as cofactors in transcriptional regulation. During neoplastic cell transformation, the proteins reveal differential expression in normal vs. tumor cells, and display intrinsic tumor promoting or tumor suppressive activities. Whereas GAP43 and MARCKS are oncogenic, tumor suppressive functions have been ascribed to BASP1 and in part to MARCKS depending on the cell type. Like MARCKS, the myristoylated BASP1 protein is localized both in the cytoplasm and in the cell nucleus. Nuclear BASP1 participates in gene regulation converting the Wilms tumor transcription factor WT1 from an oncoprotein into a tumor suppressor. The *BASP1* gene is downregulated in many human tumor cell lines particularly in those derived from leukemias, which display elevated levels of WT1 and of the major cancer driver MYC. BASP1 specifically inhibits MYC-induced cell transformation in cultured cells. The tumor suppressive functions of BASP1 and MARCKS could be exploited to expand the spectrum of future innovative therapeutic approaches to inhibit growth and viability of susceptible human tumors.

## Contribution to the Field

This review summarizes the current state of knowledge about the neuronal signaling proteins GAP43, MARCKS, and BASP1. The proteins are featured by particular effector domains, and by binding to the calcium sensor calmodulin. Besides their functions in neurons, GAP43, MARCKS, and BASP1 are also expressed in non-neuronal cells, and display intrinsic oncogenic or tumor suppressive functions. In distinct human tumors, mainly oncogenic properties are described for GAP43 and MARCKS, whereas for BASP1 a general tumor suppressive function has been reported. Genetic or pharmacological inhibition of GAP43 or MARCKS could be employed to specifically interfere with tumor growth. On the other hand, molecules mimicking the tumor suppressive function of BASP1 should represent suitable candidates for drug screening approaches in order to develop novel strategies for specific cancer treatment. Reactivation of the silenced *BASP1* gene may represent an alternative option to interfere with tumor cell viability, because endogenous *BASP1* transcription is repressed in multiple human tumor cells.

## Origin and Structures of GAP43, MARCKS, and BASP1

The so-called GMC protein class encompasses three distinct polypeptides implicated in neuronal signaling, which have been originally described under several synonyms. The GMC members are GAP43 (growth-associated protein 43, neuromodulin, or neural phosphoprotein B-50), MARCKS (myristoylated alanine-rich C-kinase substrate), and CAP-23 (cortical cytoskeleton-associated protein 23) alias NAP-22 (neuronal axonal membrane protein 22), or BASP1 (brain acid-soluble protein 1) (1–4). The GMC proteins are commonly featured by their abundant expression in axonal nerve endings. Upon local accumulation, the proteins transduce incoming signals leading to alterations in plasma membrane structure and actin cytoskeleton organization, which is required to control nerve cell growth, motility, and cell surface dynamics (3–6). Specific interactions with phospholipids and actin are implicated in axonal growth, neurotransmitter release, growth cone guidance, and synaptic plasticity, processes that are also important for memory formation and learning (1, 4, 7).

**Figure 1:**
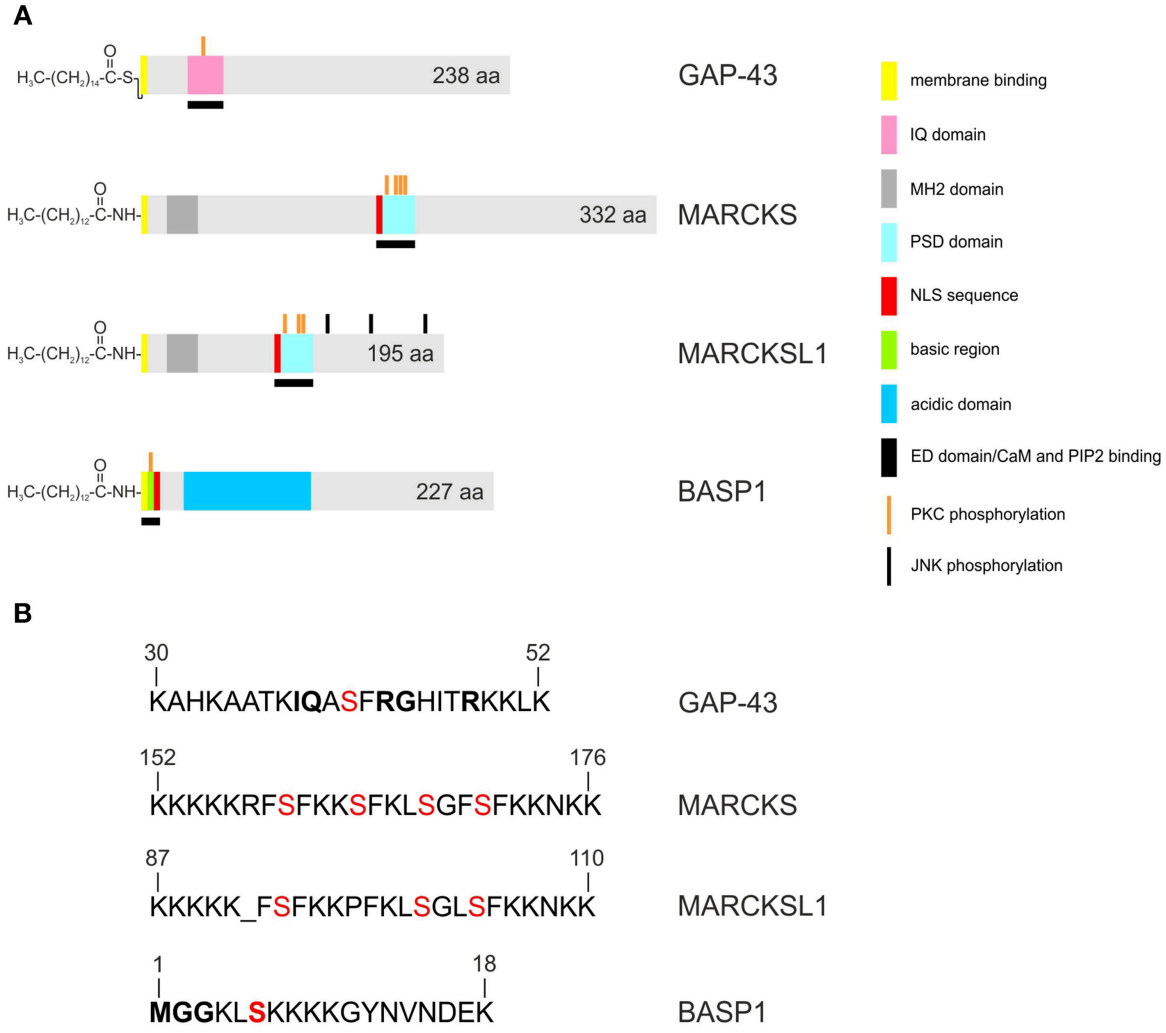
Structures of the human GAP43, MARCKS, MARCKSL1, and BASP1 proteins. Accession numbers: P17677 (GAP43), NP_002347 (MARCKS), NP_075385 (MARCKSL1), NP_001258535 (BASP1). A larger 274-amino acid isoform of human GAP43 exists (accession no. NP_001123536) **(A)** Proteins are depicted as gray bars with the indicated motifs, domains and phosphorylation sites. GAP43 is palmitoylated, and MARCKS, MARCKSL1, BASP1, are myristoylated at the N-termini. **(B)** Primary structures of the basic effector domains. Serine residues phosphorylated by protein kinase C (PKC) are in red. Conserved residues in the IQ domain of GAP43 or in the myristoylation motif of BASP1 are in bold.

**Figure 2:**
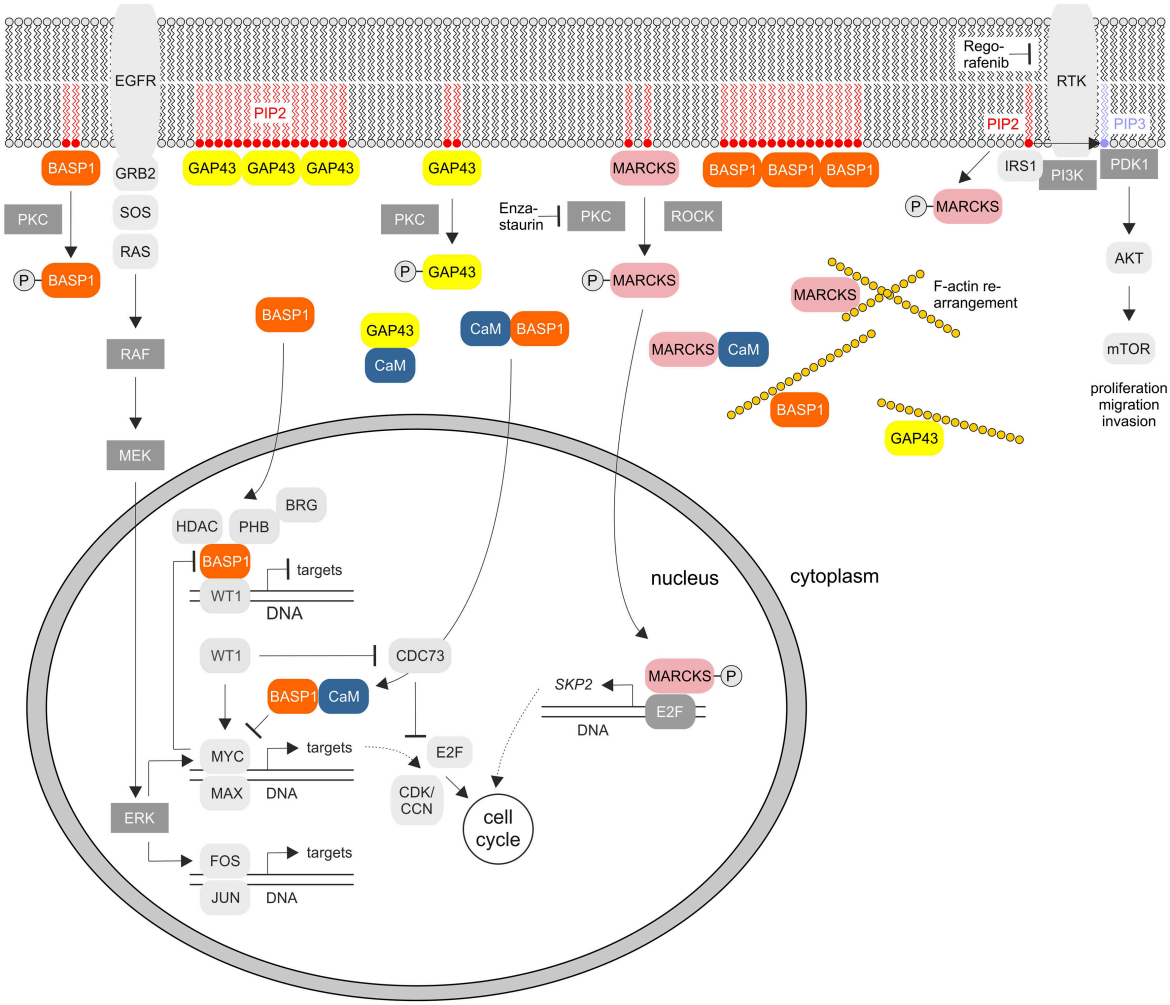
Cellular signaling pathways of the GMC proteins GAP43, MARCKS, and BASP1. The scheme summarizes the known key signaling pathways where these proteins are involved. PKC, protein kinase C; EGFR, epidermal growth factor receptor; PIP2, phosphatidylinositol-4,5-bisphosphate; PIP3, phosphatidylinositol-3,4,5-bisphosphate; GRB2, growth factor receptor bound protein 2; SOS, Ras/Rac guanine nucleotide exchange factor; ROCK, Rho associated coiled-coil containing protein kinase; RTK, receptor tyrosine kinase; IRS1, insulin receptor substrate 1; PI3K, phosphoinositide 3-kinase; PDK1, PIP3-dependent protein kinase; mTOR, mechanistic target of rapamycin kinase; MEK (MAPKK), mitogen-activated protein kinase kinase; ERK (MAPK), extracellular signal-regulated kinase; HDAC, histone deacetylase; WT1, Wilms tumor suppressor 1; PHB, prohibitin 1; CDK, cyclin-dependent protein kinase; CCN, cyclin; CDC73, cell division cycle 73; SKP2, S-phase kinase-associated protein 2. RTKs and PKC are inhibited by Regorafenib or Enzastaurin, respectively.

Although the proteins share several biochemical and biophysical properties, an initial view on the primary structures of GAP43, MARCKS, and BASP1 does not reveal significant sequence similarities (Figure 1). All three proteins have an acidic character with pI values between 4.46 to 4.64 and display unusual amino acid compositions leading to anomalous behavior in SDS-polyacrylamide gelelectrophoresis. Accordingly, the human proteins display apparent molecular masses of 57,000 (GAP43), 87,000 (MARCKS), and 52,000 (BASP1) kDa, which significantly exceeds their theoretic masses of 24,803, 31,555, and 22,693 kDa, respectively. In case of MARCKS and BASP1, the glycine residues at positions 2 are acylated with myristic acid by N-myristoyltransferase via an acid amide bonding, whereas GAP43 is linked with cysteine 3 or 4 to palmitate by palmitoyl transferase via a thioester (Figure 1). These co- or post-translational fatty acid modifications are required for association with the inner plasma membrane leaflet, and for interaction with the cortical cytoskeleton (1). Furthermore, all GMC proteins contain a basic effector domain (ED) that binds to acidic phospholipids including phosphatidylinositol-4,5-biphosphate (PIP_2_) at specialized membrane domains called lipid rafts (8). In addition, GMC proteins bind to Ca^2+^-bound calmodulin (CaM) and to actin filaments (4). All these interactions are reversibly regulated by protein kinase C (PKC)-mediated phosphorylation on serine residues present in the relevant effector domains. The phosphorylation confers negative charges onto the proteins, which then leads to their dissociation from the plasma membrane, or from CaM (5, 9, 10) (Figure 2).

Besides functional features shared between all GMC proteins, the BASP1/GAP43 and BASP1/MARCKS subsets display even more commonalities between each other regarding expression, physico-chemical properties, and biological functions (7, 11). BASP1 and GAP43 had been originally identified as major presynaptic protein components in biochemically fractionated rat brain (12). These proteins share strikingly similar roles in actin regulation, neurite outgrowth, and anatomical plasticity (2). Both sequester PIP_2_ from the plasma membrane by forming oligomers (13) (Figure 2). This is regulated by CaM, which binds to these proteins thereby disrupting the oligomeric forms and inducing displacement from the plasma membrane. In particular, GAP43 is highly expressed during neuronal outgrowth and axonal regeneration regulating actin cytoskeleton dynamics (14). High-order oligomers of BASP1 and GAP43 are intrinsically unstructured, and this disordered structure may be crucial for their function in the PIP_2_ signaling pathway (15–17). Knock-out mice containing either disrupted *GAP43* or *BASP1* are mainly non-viable pointing to essential physiological functions (2, 4, 18). On the other hand, BASP1 and MARCKS are also expressed in non-nerve tissues like kidney, testis, and lymphoid cells (4, 7, 13, 19). Besides shuttling between plasma membrane and cytoplasm, BASP1 and MARCKS are localized in the nuclei of distinct cell types where they are involved in gene regulation (Figure 2). The nuclear import of MARCKS or BASP1 is mediated by nuclear localization motifs present in their effector domains (20, 21) (Figure 1).

## GAP43 Is Implicated in Oncogenesis

**Figure 3:**
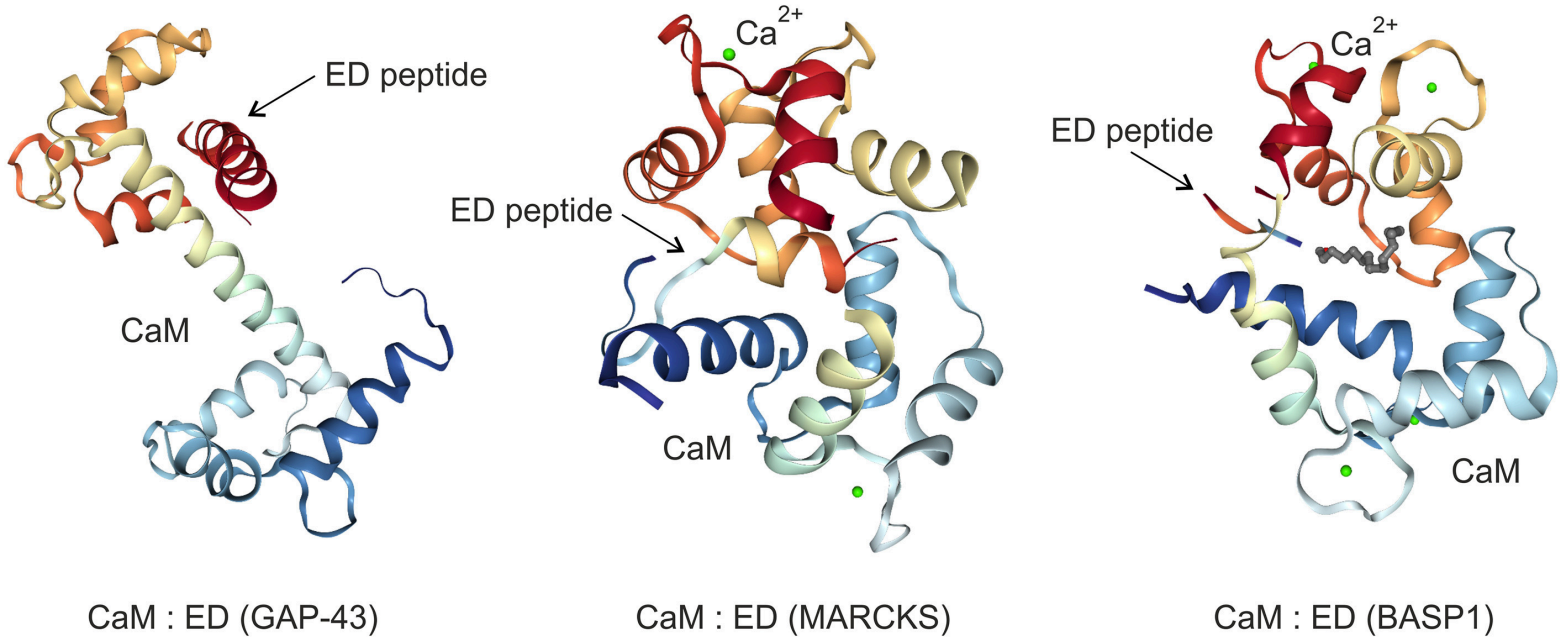
Crystal structures of the GAP43, MARCKS, and BASP1 effector domains (ED) bound to the calmodulin (CaM) protein in the absence (GAP43) or in the presence (MARCKS, BASP1) of calcium ions (Ca^2+^). The images were created from the protein data bank (PDB) entries 4E53 (27), 1IWQ (44), 1L7Z (6), respectively, and adapted.

The palmitoylated GAP43, originally termed growth or plasticity protein is essential for neuronal pathfinding and highly expressed in neuronal growth cones (22, 23). The protein is thus associated with nerve growth and represents a major component of the motile “growth cones” that form the tips of elongating axons (24). GAP43 overexpression induces neuronal outgrowth (25), and knock-out mice lacking GAP43 due to homologous recombination die in the early postnatal period with retinal axons trapped in the optic chiasm (18). The interaction with Ca^2+^-free CaM (apo-CaM), which is crucial for learning and memory formation, occurs via the IQ domain of GAP43 representing the so-called effector domain (Figure 1). This domain contains a protein motif with the consensus sequence IQ-(X)_3_-RG-(X)_3_-R (26) that is intrinsically unstructured. Upon binding to the central α-helix of apo-CaM, the IQ domain adopts an α-helical conformation. This structure was determined by X-ray diffraction analysis (27) and shows that the IQ domain is orientated in an almost perpendicular manner toward the CaM protein (Figure 3). PKC-mediated phosphorylation of serine 41 within the IQ domain leads to its dissociation from CaM, which is otherwise bound in a negatively charged pocket of apo-CaM containing asparate and glutamate residues (27). Besides CaM, several proteins have been identified, which interact with GAP43 in neuronal cells like the axonal protein dihydropyrimidinase-like 3 (DPYSL3) (28), or the microtubule-associated protein MAP-2 (29).

The first link of GAP43 to neoplastic cell transformation was obtained by differential gene expression analysis. GAP43 was found to be strongly up-regulated in chicken embryo fibroblasts transformed by several oncogenes (30) suggesting that associated perturbations in PKC- or Ca^2+^/CaM-mediated signal transduction pathways are implicated in oncogenesis. In fact, CaM is involved in the promotion of physiological cell cycle progression but also in tumorigenesis (31–33). GAP43 is induced by neutrophin receptor stimulation and drives neuronal progenitor cell migration but is also activated in malignant peripheral nerve sheath tumors (34, 35). Furthermore, GAP43 expression is induced in various brain tumor cell lines, where it is highly expressed in axonal growth cones (23, 35). A tumor promoting function has been ascribed to highly-expressed GAP43 in human glioblastomas, where it is involved in the generation of so-called tumor microtubes (36). These ultra-long membrane protrusions are a typical feature of astrocytic brain tumors. Microtubes have a high content of F-actin and thus ressemble membrane nanotubes. However, microtubes are longer, thicker, and more stable. In brain tumors, they are used as routes for invasion and proliferation. Connexin 43 gap junctions allowing multicellular communication are a feature of microtubes. Tumor types forming such multicellular network structures include glioblastomas and represent so far incurable neoplasms. In fact, therapy resistance of astrocytomas depends on the interconnection of these ultra-long tumor microtubes, thereby preventing local accumulation of high calcium ion concentrations. This is necessary because increased Ca^2+^ levels would induce apoptosis in glioma cells, which is on the other hand a prerequisite for radiotherapy-induced cytotoxicity (37). Genetic knock-downs of *GAP43* in astrocytomas show abnormal tumor microtube formation and impaired tumor cell dissemination (36). Furthermore, *GAP43* inactivation led to reduced microtube connections and gap junctions, resulting into significantly smaller tumors in mouse brains. This is explained by the fact that GAP43 induces tumor microtube formation just as it triggers axon generation in neurons. In summary, GAP43 drives microtube-dependent tumor cell invasion, proliferation, interconnection, leading to resistance toward radiotherapy-induced cell death (36).

## MARCKS: a Dichotomic Role in Oncogenesis and Tumor Suppression

MARCKS, a substrate of the protein kinases PKC and ROCK (38), is ubiquitously expressed as a rod-shaped protein. The protein is implicated in cell migration, proliferation, and survival (39), and translocates between cell membrane and cytoplasm triggered by reversible phosphorylation and binding to CaM (Figure 2). MARCKS sequesters PIP_2_ via its effector domain, and cross-links actin filaments thus dynamically altering cytoskeleton and cell architecture with an impact on cell motility, phagocytosis, membrane trafficking, and mitogenesis (40). MARCKS is also shuttled into the nucleus mediated by the effector domain containing a nuclear localization signal (Figure 1). Thereby, nuclear PIP_2_ levels increase suggesting a critical role of the MARCKS effector domain in order to control PIP_2_ levels, nuclear localization, and possibly gene expression (21). The N-terminus of MARCKS contains the myristoylation site MGAQFS in accordance with the consensus sequence MGXXX^S^/_T_ (41), and a so-called MH2 domain (Figure 1) with a serine phosphorylation site whose putative protein binding capabilities are yet unknown (40). The acidic MARCKS protein is intrinsically unstructured even when complexed with CaM. Phosphorylation of the effector domain displaces MARCKS from the membrane, and this post-translational modification inhibits the F-actin cross-linking activity of MARCKS (42, 43). Analyzing the crystal structure between a peptide encompassing the effector domain and Ca^2+^-bound CaM revealed that this domain adopts an elongated structure with a short α-helical region interacting with the C-terminal lobe of CaM but not within the hydrophobic pocket of the N-terminal lobe (44) (Figure 3).

Emerging evidence implies that *MARCKS* functions as an oncogene playing a critical role in cancer development, progression, and metastasis (40). There are multiple reports describing the MARCKS protein being associated with invasion in glioblastoma, cholangiocarcinoma, leukemia, melanoma, ovarian cancer, prostate cancer, and inflammatory breast cancer (21, 45–51). Furthermore, this protein exhibits a fundamental role in mediating chemoresistance of breast and lung cancer cells (46). Highly expressed MARCKS contributes to stromal cancer-associated fibroblast activation and promotes ovarian cancer metastasis, which is associated with poor patient survival. Likewise, MARCKS is upregulated in kidney cancer, and genetic or pharmacologic MARCKS suppression leads to a decrease in cell proliferation and migration of renal carcinoma cells (39). Pharmacologic MARCKS suppression was achieved using a 24-amino acid MARCKS-specific peptide identical to the myristoylated MARCKS N-terminus (39, 52). Application of the myristoylated peptide reduces the metastatic potential of non-small-cell lung cancer cells (NSCLC), in which MARCKS is expressed at elevated levels (46). Thereby, this peptide reduces MARCKS serine 159 phosphorylation within the effector domain possibly by acting as a competitive inhibitor. Normally, membrane-bound MARCKS is phosphorylated by PKC or ROCK, which then leads to membrane dissociation and F-actin rearrangement (Figure 2). Membrane PIP_2_ becomes liberated from MARCKS and is phosphorylated to PIP_3_ by phosphatidylinositol-3-kinase (PI3K). This leads subsequently to activation of the AKT pathway thereby promoting invasion and cell migration (46) (Figure 2). Presumably, only the previously membrane-bound MARCKS but not the peptide is able to activate the PI3K pathway. This particular subcellular localization and the myristoylation of MARCKS are both required to promote cell migration and invasion. High levels of the myristoylated MARCKS-specific peptide binding to the membrane therefore competitively prevents full-length MARCKS binding and subsequent AKT activation (46).

Paradoxically, in transformed fibroblasts and some distinct highly proliferating melanoma, neuroblastoma, glioma, or colorectal cancer cells, MARCKS is downregulated (53–56). In prostate cancer, the *MARCKS* down-regulating microRNA-21 (miR-21) is overexpressed, and selective miR-21 suppression results in apoptosis sensitivity, and cell invasion inhibition (57). Moreover, certain inactivating MARCKS mutations lead to adenocarcinoma (58), suggesting that MARCKS has a dichotomic role in oncogenesis and tumor suppression depending on the cancer cell type (40). For instance, in colorectal cancers, MARCKS is a preferential target of mutational inactivation in tumors, which is associated with an adverse prognosis (55). Inactivation of MARCKS in colon cancer cells has anti-apoptotic effects resulting finally in decreased caspase-3 and caspase-8 activities. In parallel, pro-survival pathways become activated, thus increasing AKT phosphorylation (55). Possibly, phosphorylation of MARCKS is critical for the conversion from a tumor suppressor into an oncoprotein (40). Phosphorylated MARCKS was identified in the nucleus as a cofactor of the transcription factor E2F-1 to regulate transcription of S-phase kinase associated protein 2 (SKP2), so that cell cycle progression is promoted (59) (Figure 2). Hence, MARCKS appears to be a phosphorylation-controlled negative regulator of signaling pathways that is dysregulated in cancer, which could be exploited for the design of appropriate drugs targeting MARCKS (40).

Besides MARCKS, a related protein termed MARCKS-like protein 1 (MARCKSL1, MLP, MRP) exists, which is also implicated in cancerogenesis (60–63). MARCKSL1 is significantly shorter than the MARCKS protein but has a similar topography concerning its primary structure (Figure 1). Similar to MARCKS, MARCKSL1 has a variety of functions in embryonic development, brain plasticity, inflammation, and regeneration. MARCKSL1 also dissociates from the membrane into the cytosol upon PKC-mediated phosphorylation or CaM binding, thereby modulating actin dynamics and vesicular trafficking (63). MARCKSL1 binds to F-actin via its conserved effector domain but this does not lead to F-actin cross-linking in contrast to the MARCKS paralogue (62, 64). However, phosphorylation by Jun N-terminal protein kinase (JNK) on three C-terminal residues (Figure 1) enables MARCKSL1 to bundle and to stabilize F-actin leading to cell migration inhibition, whereas dephosphorylation of these C-terminal residues induces cell migration. In a broad range of cancer types MARCKSL1 is upregulated where is has a critical role in cell migration (62). Like MARCKS, MARCKSL1 is highly expressed in distinct human brain tumor cell lines (see below).

## BASP1, a Potential Tumor Suppressor Linked to Transcriptional Regulation

The 23-kDa acidic BASP1 protein was originally isolated as a membrane and cytoskeleton-associated protein from rat and chicken brain (5, 65). It is particularly abundant in nerve terminals during brain development and implicated in neurite outgrowth, maturation of the actin cytoskeleton, and organization of the plasma membrane. BASP1 is also expressed in various other tissues (10, 20, 66) but its precise biochemical and biological functions are still unknown. In most cases, homozygous *BASP1* knock-out leads to embryonal lethality. Only about 10% of the knock-out mice survive to adulthood but these animals exhibit a complex phenotype including deficient production of induced nerve sprouting at the adult neuromuscular junction (2).

**Figure 4:**
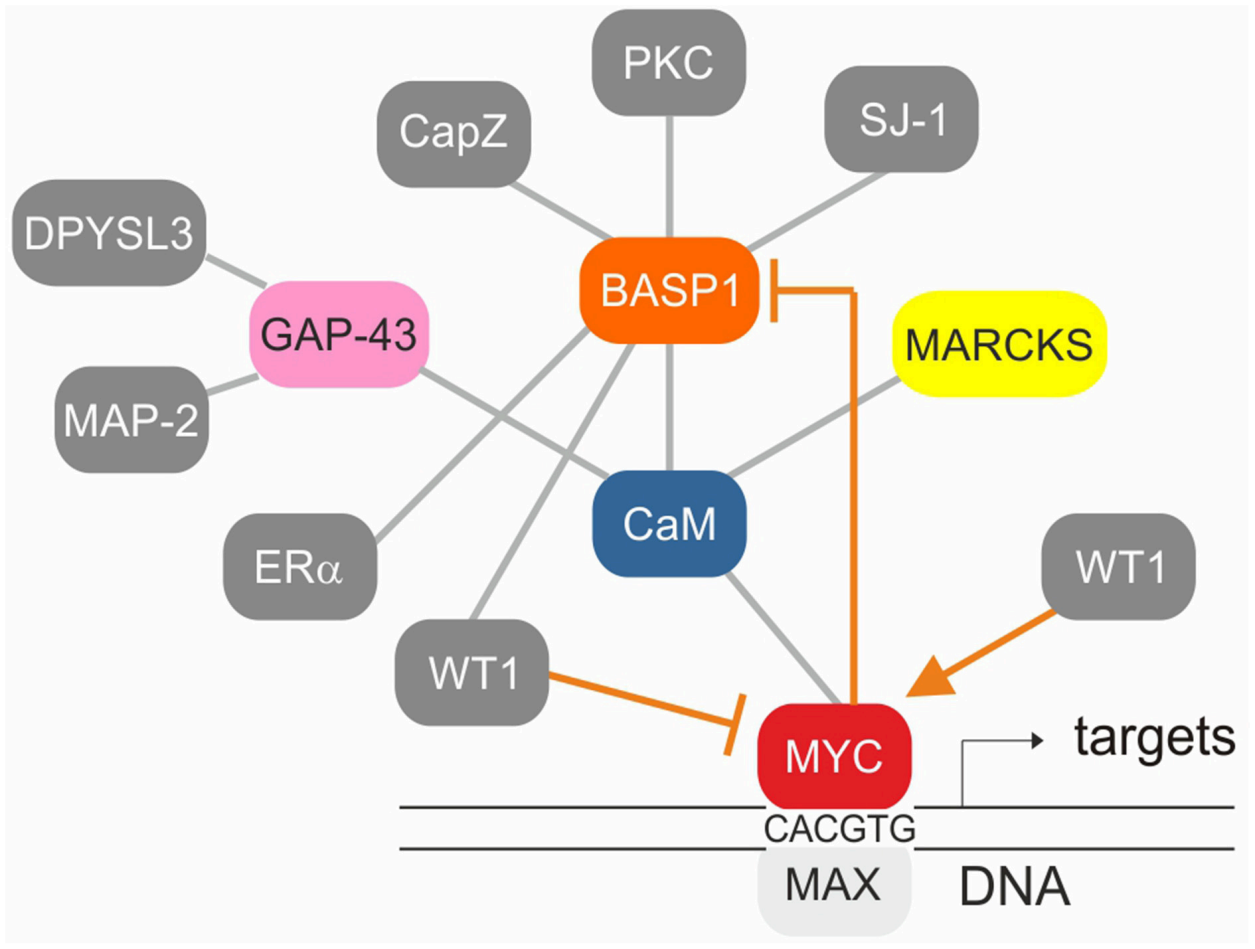
Established protein-protein interactions (gray) and transcriptional regulation connections (orange) within the BASP1/CaM/MYC network. The MYC/MAX heterodimer binds to canonical motifs (E-boxes) present in the promoter regions of numerous MYC target genes. WT1 stimulates whereas WT1/BASP1 represses *MYC* transcription. MYC/MAX on the other hand represses *BASP1* transcription thereby preventing its own repression by WT1/BASP1.

Myristoylated BASP1 binds to CaM (5, 67), is a substrate of PKC, and shares distinct biochemical and biophysical properties with the cytosolic growth-associated proteins GAP43 and MARCKS (5, 6, 10, 67) described above. Likewise, phosphorylation by PKC leads to disruption of BASP1/membrane lipid and BASP1/CaM interactions (9, 67) (Figure 2). Besides, several cytoplasmatic BASP1-binding proteins have been identified like the actin capping protein (Cap Z) (68), or the PIP_2_ phosphatase synaptojanin-1 (SJ-1) (69) (Figure 4). Nuclear translocation of BASP1 is mediated by a bipartite nuclear localization sequence (NLS) positioned in the N-terminus (20, 70, 71) (Figure 1). The N-terminal region contains a serine residue at position 6, which is phosphorylated by PKC. This post-translational modification leads to disruption of the BASP1 interaction with lipids and with CaM (4, 9). BASP1 attenuates the transcriptional activity of the Wilms' tumor suppressor protein (WT1) acting as a cosuppressor (20, 70) (Figure 2). WT1, a zinc-finger protein, represents a major gene regulator important for cell growth, apoptosis, and differentiation, and plays an essential role in urogenital development and malignancy. Although WT1 has been originally identified as a tumor suppressor (20, 70), it is also overexpressed in a variety of human cancers, including acute leukemias (72–74) and solid tumors like breast cancer. In these diseases, expression of WT1 is associated with a poor prognosis. WT1 is negatively regulated by a BASP1-binding suppression region present in the transcriptional activation domain (20). Oncogenic and tumor suppressive activities are thus modulated by nuclear BASP1 upon physical interaction with WT1 (20, 75) (Figure 2). WT1-repression by BASP1 is also relevant during reprogramming of differentiated cells into induced pluripotent stem cells. Usually, this process is achieved by exogenous transcription factors such as MYC or Sox-2. On the other hand, signaling pathways initiated by proteins associated with the cell membrane like BASP1 can drive differentiation. Inhibition of membrane-associated BASP1 by an antibody has the same effect as Sox-2 overexpression and suffices to reprogram cells to a pluripotent state. Thereby, crucial nuclear factors like WT1 become derepressed (76).

Nuclear BASP1 is also bound by PIP_2_, which then enables an interaction with histone deacetylase (HDAC). This leads to subsequent transcriptional repression (75). In addition, the transcriptional repressor prohibitin (PHB) was identified as a component of the WT1-BASP1 transcriptional complex (71). Prohibitin interacts with BASP1 leading to recruitment of the chromatin remodeling factor BRG to WT1-responsive promoters resulting in dissociation of the coactivator CBP from the promoter region of WT1 target genes (Figure 2). In fact, during epithelial-mesenchymal or mesenchymal-epithelial transition, WT1 either recruits CBP and p300 as coactivators, or BASP1 as corepressor, respectively (77). Like BASP1, prohibitin is associated with phospholipids. The recruitment of PIP_2_ and HDAC1 to WT1 target genes is therefore dependent on the concerted activities of BASP1 and prohibitin (71).

Multiple genes regulated by the WT1/BASP1 complex have been identified (66, 74). One of them is *MYC* being activated by WT1, and suppressed by the WT1/BASP1 complex (66, 70, 78, 79) (Figure 2). On the other hand, transcription of the *BASP1* gene is specifically repressed in v-*myc*-transformed avian cells, and ectopically expressed BASP1 inhibits v-*myc*-induced oncogenesis (80). BASP1 does not physically interact with v-Myc, but v-Myc binds to the BASP1-interaction partner CaM (81) suggesting a functional connection between these three proteins (Figure 4). Increased CaM levels enhance the transcriptional and oncogenic v-Myc activities (81) suggesting that excess BASP1 may interfere with the v-Myc/CaM interaction, and consequently with central v-Myc/CaM functions.

*BASP1* is also downregulated in multiple mammalian tumors like carcinoma, acute, and chronic lymphocytic leukemia (ALL, CLL), or melanoma by direct transcriptional repression, microRNA-guided downregulation, or promoter methylation (82–88). In addition, *BASP1* is downregulated in lung cancer where the oncogenic microRNA miR-191 is upregulated. In these tumor cells *BASP1* suppression is caused by specific miR-191-mediated mRNA degradation (83). In mouse, *BASP1* is downregulated, among several other anti-cancer genes, in induced cutaneous squamous cell carcinoma by the concomitantly activated long non-coding RNA AK144841 (89). Recently, tumor-suppressive functions of BASP1 have been also observed in several human cancer models:

- Ectopic BASP1 expression in thyroid cancer cell lines inhibits their growth as well as tumor formation in xenografts (90).
- BASP1 binds to the estrogen receptor α (ERα) (Figure 4) and acts as a transcriptional corepressor thus enhancing the effect of the estrogen antagonist tamoxifen (91). BASP1 elicits tumor suppressor activity in breast cancer and BASP1 expression levels correlate with increased patient survival (91). In this context, it is interesting to observe that the BASP1-interaction partner CaM induces ERα dimerization, and that disruption of the ERα/CaM interaction could represent a potential therapeutic strategy for targeting ERα-positive breast cancers (31).
- Methylation-associated silencing of *BASP1* contributes to leukemogenesis in acute myeloid leukemia (AML). Ectopic BASP1 expression inhibits proliferation and colony formation of AML cell lines by inducing apoptosis and cell cycle arrest (92).

**Figure 5:**
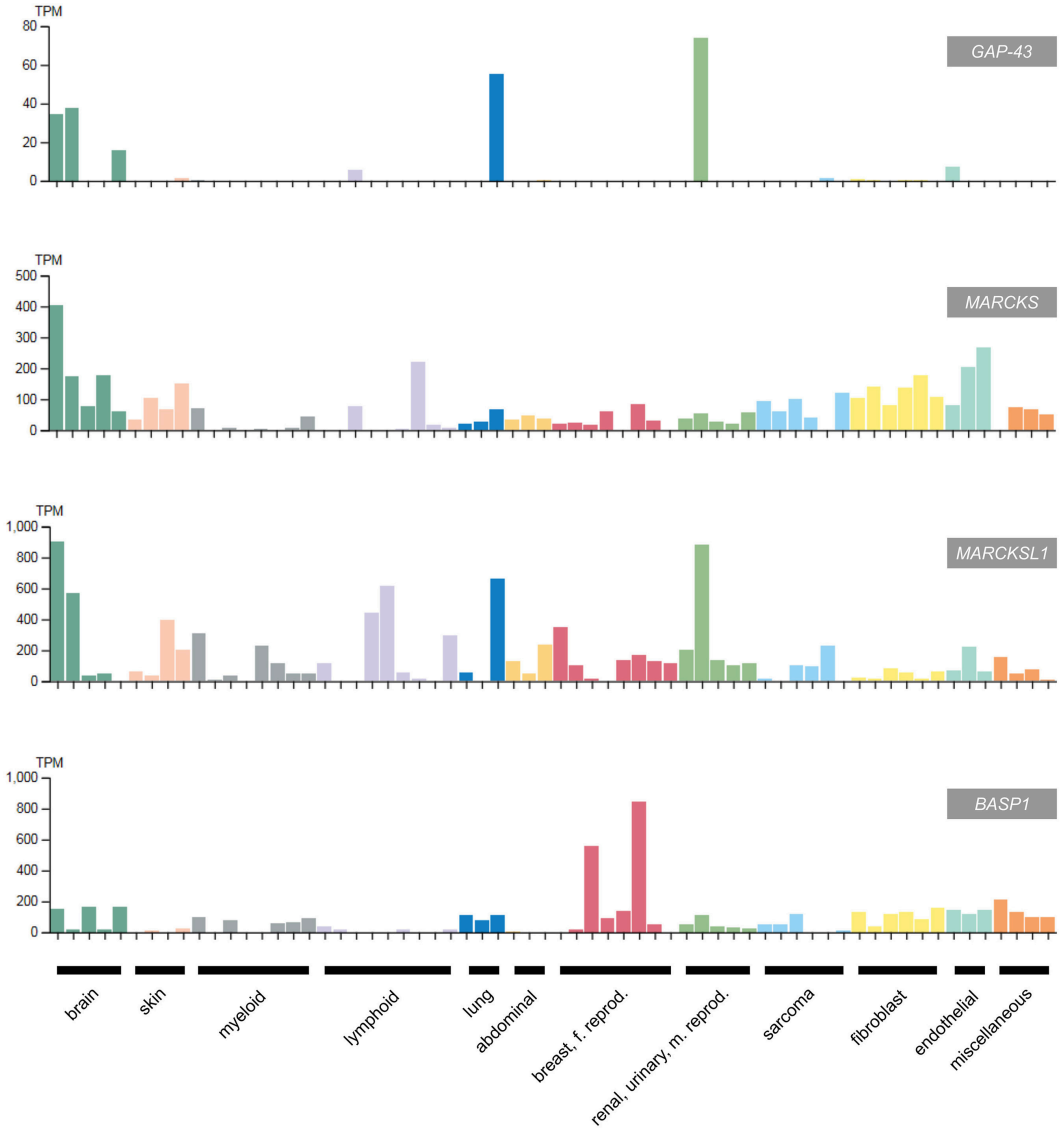
Expression of *GAP43, MARCKS, MARCKSL1*, and *BASP1* in selected human cancer cell lines classified according to the original cancer type (93) (adapted from *www.protein-atlas.org* version 18). RNA expression levels determined by RNA-Seq are given in transcripts per kilobase million (TPM). *GAP43* and the *MARCKS* genes are highly expressed in brain tumor cell lines, whereas *BASP1* is suppressed in most cancer cell lines apart from the cervical tumor cell lines HeLa and SiHa. The following cell lines were assessed: brain (AF22, SH-SY5Y, U138-MG, U-251MG, U-87MG), skin (A-431, HaCaT, SK-MEL-30, WM-115), myeloid (HAP1, HEL, HL-60, HMC-1, K-562, NB-4, THP-1, U-937), lymphoid (Daudi, HDLM-2, Karpas-707, MOLT-4, REH, RPMI-8226, U266/70, U-266/84, U-698), lung (A549, HBEC3-KT, SCLC-21H), abdominal (CACO-2, CAPAN-2, Hep G2); breast, female reproductive system (AN3-CA, EFO-21, HeLa, hTERT-HME1, MCF7, SiHa, SK-BR-3, T-47d), renal, urinary, male reproductive system (HEK 293, NTERA-2, PC-3, RPTEC TERT1, RT4), sarcoma (ASC diff, ASC TERT1, LHCN-M2, RH-30, U-2 OS, U-2197), fibroblast (BJ, BJ hTERT+, BJ hTERT+ SV40 Large T+, BJ hTERT+ SV40 Large T+ RasG12V, fHDF/TERT166, HBF TERT88), endothelial (hTEC/SVTERT24-B, HUVEC TERT2, TIME), miscellaneous (BEWO, HHSteC, HSkMC, hTCEpi).

Accordingly, *BASP1* is downregulated in most human tumors and tumor cell lines, except of distinct cervical cancer cells where *BASP1* levels are paradoxically high (Figure 5). In this cell type, amplification of the *BASP1* locus (chromosome 5p15.1) has been reported, which could explain the high expression level as consequence of increased gene dosis (94). Furthermore, in distinct cervical cancer cells BASP1 even promotes tumor growth (95). Several cervical cancer cell lines contain high *BASP1* but low *WT1* and *MYC* levels, suggesting that the growth-inhibiting function of BASP1 is restricted to tumor cells with characteristic features, for instance aberrantly elevated *WT1* and *MYC* expression levels, as it is the case in most lymphoma cells (www.proteinatlas.org) (93). In this context, it is of interest to note that there is a non-coding mRNA encoded by a human *BASP1* pseudogene on chromosome 13 (accession no. NR033774), and a related *BASP1* mRNA encoding a BASP1-like protein with a size of 173 amino acids (accession no. BAG58770).

Besides inhibition of transcriptional activation and cell transformation, BASP1 also acts as a pro-apoptotic factor in diabetic nephropathy and in albumin-induced programmed cell death (96, 97). This is reminiscent to MARCKS, which occasionally acts as a tumor suppressor and mediator of apoptosis in a variety of human neoplasms like colorectal cancers (see above). In summary, BASP1 is involved in multiple biological processes, which all require interactions with a variety of distinct proteins. This is certainly facilitated by the highly flexible protein structure of BASP1 due to its intrinsic disorder (15, 17). In fact, BASP1 belongs to a class of proteins that have no defined tertiary structure. These proteins display conformational plasticity and are usually featured by a low amount of bulky hydrophobic amino acid residues and a high content of charged and hydrophilic residues (98, 99). Using a novel bioinformatic protein-protein interaction prediction tool, known interaction partners of BASP1 have been confirmed, and several new potential ones were identified such as histone deacetylase 1 (HDAC1), actin beta (ACTB), caspase-3 (CASP3), nucleoplasmin (NPM), or the progesterone receptor (PGR) (98).

## Are the GMC-Class Proteins Suitable Drug Targets?

Due to their particular oncogenic or tumor-suppressive functions, individual GMC proteins may represent specific molecular targets or templates for the design of possible drugs either in form of small organic molecules, short peptides, or interfering RNAs for potential future therapeutical applications.

### GAP43

Specific targeting of GAP43, which is implicated in astrocytic brain tumorigenesis by promoting microtube formation and tumor cell invasion, could favor astrocytoma cell disconnection, thereby reducing the obstructive treatment resistance of these glioblastomas (36). Therefore, targeting their ultra-long microtubes leading to disconnection of astrocytoma cells could emerge as a new therapeutic principle to reduce treatment resistance of this disease. Genetic knock-down of *GAP43* led to deficiencies in tumor-microtube formation causing a distinct reduction of tumor size in the mouse brain and to an improved survival of the animals (36). Therefore, screening for potential GAP43 inhibitors could lead to novel compounds specifically targeting GAP43 in these so far incurable neoplasms.

### MARCKS

Phosphorylation of MARCKS in the effector domain is crucial for its oncogenic function. There are elevated levels of phosphorylated MARCKS in highly invasive non-small-cell lung cancer cells, in drug-resistant myeloma, or in kidney cancer cells (39, 46, 100). Application of a 25-amino acid peptide (MANS), identical to the MARCKS N-terminus reduced MARCKS phosphorylation leading to impaired cell migration and reduced metastatic potential in lung cancer cells (46). The peptide induces cyctotoxicity also on multiple myeloma cells, an effect that was enhanced by the synergistically acting proteasome inhibitor bortezomib. Administration of MANS leads to cell cycle arrest and growth-suppression due to p27^KIP1^ accumulation and reduced AKT1 phosphorylation. In high-grade renal cell carcinoma, genetic *MARCKS* inhibition by short interfering RNA or pharmacological inhibition by MANS leads to decreased cell proliferation and migration (39). As outlined above, MARCKS acts upstream of the AKT/mTOR pathway (Figure 2). After *MARCKS* knock-down in renal cell carcinoma cells, the efficacy of the tyrosine kinase inhibitor Regorafenib was enhanced, whereas MARCKS attenuation by MANS led to inactivation of AKT and mTOR. This leads to a decrease of kidney cancer cell survival, and demonstrates the potential of MARCKS as a possible druggable therapeutic target (39, 40).

Another strategy to inhibit MARCKS phosphorylation is the application of the macrocyclic bisindolylmaleimide Enzastaurin, a PKC inhibitor significantly enhancing the sensitivity of drug-resistant cells toward the proteasome inhibitor bortezomib and other anti-myeloma drugs. Thereby Enzastaurin competes with ATP for the nucleotide-binding site of PKC and blocks its activation (100). However, clinical phase III trials performed later showed that Enzastaurin alone failed to prolog the lifespan of the patients participating in this study (101) indicating that additional targets have to be attacked in order to overcome cancer remission. Therefore, combination of a MARCKS and a proteaseome inhibitor could represent a basis to develop novel myeloma therapies.

### BASP1

In several human cell types, BASP1 has a tumor-suppressive function (90–92). Furthermore, in multiple carcinoma, melanoma, and leukemia, *BASP1* transcription is silenced by promoter methylation (82, 84, 92), a typical DNA modification in the regulatory regions of tumor suppressors in cancer. In v-*myc*-transformed avian cells, *BASP1* is directly repressed by the transcription factors SP1 and MYC at the promoter level (80) (Figures 2, 4). Because the orthologous human *BASP1* promoter has a similar structure, and *BASP1* is downregulated in a variety of human leukemia cells containing elevated *MYC* levels (102), human *BASP1* transcription may be repressed by a similar mechanism. MYC is one of the most frequently deregulated oncogenes in many cancer types and a hallmark of the majority of human cancers (103–105). In acute lymphoblastic and myeloid leukemia (ALL, AML), *MYC* is often overexpressed and frequently associated with disease progression (106). All members of the MYC protein family physically interact with the calcium sensor calmodulin in a Ca^2+^-dependent manner (81) and in T-cell lymphoma, overexpressed MYC is stabilized by Ca^2+^/calmodulin-dependent protein kinase II γ (CAMKIIγ) phosphorylation (107). Several human leukemia-derived cell lines have been used as a model system to study MYC functions (106) and all these cells contain high *MYC* and low *BASP1* levels (www.proteinatlas.org) (93). Furthermore, ectopic expression of human BASP1 inhibits proliferation and colony formation in AML cells thus leading to apoptosis and cell cycle arrest (92).

In lung cancer cells, *MYC* is a transcriptional target of oncogenic WT1, which enhances proliferation and impedes apoptosis (78) (Figures 2, 4). Overexpression of WT1 induces a significant increase in the abundance of endogenous MYC protein in breast cancer cells (79), whereas a WT1/BASP1 complex represses the *MYC* promoter (70). Oncogenic WT1 transcriptionally represses the tumor suppressor CDC73 encoding an RNA polymerase II interactor (Figure 2). CDC73 is part of a transcriptional regulatory complex, which on the other hand represses oncogenes like *MYC* or *CCND1* (cyclin D1) (108).

Due to the established protein-protein interactions of WT1:BASP1, BASP1:CaM, and CaM:MYC (Figure 4), oncogenic MYC functions may be influenced not only by BASP1 but also by BASP1 analoga. In fact, mutational analysis of the highly conserved BASP1 effector domain has revealed that the CaM binding capacity and the transformation inhibition potential correlate with each other (6, 80). Accordingly, a small peptide representing the BASP1 effector domain could interfere with the proliferation of human cancer cells in which *MYC* is highly activated. In this context, the BASP1 effector domain would act like a CaM inhibitor similar to the compounds trifluoperazine (TFP), or W-7. These small organic molecules bind tightly to the hydrophobic CaM pockets as the BASP1 effector domain peptide does (6, 109, 110). Calcium-dependent conformational changes of calmodulin have been also observed upon binding to melittin (MEL), a 26-amino acid amphipatic peptide component of bee venom with structural similarties to the BASP1 effector domain (111, 112). MEL has offered a variety of promising anti-cancer effects although further optimization approaches are required in order to lower its non-specific cytotoxicity (112). MEL can induce apoptosis of hepatocellular carcinoma cells by activating Ca^2+^/calmodulin-dependent protein kinase, transforming growth factor-beta-activated kinase 1 (TAK1), and the JNK/p38 MAPK pathway (112).

## Outlook

In summary, novel compounds based on the templates of tumor-suppressive GMC proteins, or molecules specifically interfering with the functions of oncogenic GMC proteins could contribute to the development or refinement of adequate cancer therapies. During the last decades, cancer treatment has evolved more and more from non-specific cytotoxic agents to pathway-selective therapeutical approaches based on distinct molecular mechanisms in signal transduction. These targeted molecular therapies are focused to induce changes in metabolism and physiology of tumor cells. In addition, specific immunotherapies have been developed strengthening the immune system of the organism in order to selectively eliminate malignant cells. Combination of these two pillars in oncologic treatment, key molecule-targeted approaches and immunotherapy could synergistically enhance specificity and efficacy (113). Immunotherapy using specific humanized antibodies enhances the host immune system by overcoming so-called checkpoints like the cyctotoxic T lymphocyte-associated molecule-4 (CTLA-4) or the programmed cell death receptor-1 (PD-1) (114, 115). In addition, chimeric monoclonal antibodies and antibody drug conjugates (ADC) combining monoclonal antibodies with a cytotoxic agent are employed to promote cancer cell destruction, using genetically modified T-cells and vaccines in form of modified dendritic cells that express tumor peptide antigens (114, 115).

A potential link between the above described GMC protein network (Figure 4) and immunotherapeutical strategies has been recently demonstrated with the WT1 oncoprotein. WT1 is an attractive target for cancer therapy because it is overexpressed in a wide range of leukemias and solid tumors, whereas in normal tissues it is expressed at low levels and negatively regulated by BASP1 (74). Chimeric antigen receptor (CAR)-T cells with antitumor activity have been developed by targeting processed surface peptides on cancer cells derived from the intracellular WT1 protein (116). CAR-T cells with a chimeric antigen receptor consisting of a single variable WT1-specific antibody fragment in complex with a major histocompatibility (MHC) protein were tested using a xenograft model. The antitumor effect of these CAR-T cells was further enhanced by vaccination with dendritic cells loaded with the corresponding antigen (116). Using subsidiary immunotherapeutical approaches to specifically target key molecules in carcinogenesis could therefore substantially expand the current options to treat cancer.

## Author Contributions

MH selected the topic and designed the outline of the review. RS analyzed protein domains in GAP43, MARCKS, and BASP1. MH and RS wrote the article.

### Conflict of Interest Statement

The authors declare that the research was conducted in the absence of any commercial or financial relationships that could be construed as a potential conflict of interest.
